# Validation of a New Test for *Schistosoma haematobium* Based on Detection of *Dra*1 DNA Fragments in Urine: Evaluation through Latent Class Analysis

**DOI:** 10.1371/journal.pntd.0001464

**Published:** 2012-01-03

**Authors:** Olufunmilola Ibironke, Artemis Koukounari, Samuel Asaolu, Irini Moustaki, Clive Shiff

**Affiliations:** 1 Department of Molecular Microbiology and Immunology, Johns Hopkins University, Baltimore, Maryland, United States of America; 2 MRC Centre of Outbreak Analysis and Modeling, Department of Infectious Disease Epidemiology, Imperial College London, St. Mary's Campus, London, United Kingdom; 3 Department of Zoology, Obafemi Awolowo University, Ile Ife, Nigeria; 4 Department of Statistics, London School of Economics, London, United Kingdom; Natural History Museum, London, United Kingdom

## Abstract

**Background:**

Diagnosis of urogenital schistosomiasis in chronically infected adults is challenging but important, especially because long term infection of the bladder and urinary tract can have dire consequences. We evaluated three tests for viable infection: detection of parasite specific DNA *Dra*1 fragments, haematuria and presence of parasite eggs for sensitivity (Se) and specificity (Sp).

**Methods:**

Over 400 urine specimens collected from adult volunteers in an endemic area in Western Nigeria were assessed for haematuria then filtered in the field, the filter papers dried and later examined for eggs and DNA. The results were stratified according to sex and age and subjected to Latent Class analysis.

**Conclusions:**

Presence of *Dra*1 in males (Se = 100%; Sp = 100%) exceeded haematuria (Se = 87.6%: Sp = 34.7%) and detection of eggs (Se = 70.1%; Sp = 100%). In females presence of *Dra*1 was Se = 100%: Sp = 100%, exceeding haematuria (Se = 86.7%: Sp = 77.0%) and eggs (Se = 70.1%; Sp = 100%). *Dra*1 became undetectable 2 weeks after praziquantel treatment. We conclude detection of *Dra*1 fragment is a definitive test for the presence of *Schistosoma haematobium* infection.

## Author Summary

The definitive test for schistosomiasis has been to detect parasite eggs in excreta. This is effective in children but as people age, it is difficult to find the eggs in spite of suspicion of infection. It also implies that adults have thrown off the infection, and therefore the infection is mainly one of children. However this is not the case as adults with chronic infections can develop severe bladder infection that can progress to bladder cancer. In clinical situations a sensitive test for urogenital schistosomiasis is important. We have recently shown that it is possible to detect schistosome species-specific DNA in urine, even in the absence of eggs in excreta. Here we use latent class analysis to compare sensitivity and specificity of three test procedures, haematuria, eggs in urine and specific DNA in urine in the absence of a gold standard. We show that the latter test is extremely promising for the diagnosis of urogenital schistosomiasis and should also be useful in field studies as it will be able to reveal infections in people frequently presumed to be uninfected. This new test indicated the presence of schistosomes in 10.7% of adults who did not pass eggs in urine specimens.

## Introduction

*Schistosoma haematobium* (the urogenital type of schistosomiasis) is endemic throughout the African continent as well as parts of Western Asia. It is a blood borne trematode parasite which in the adult form lives in the capillary plexus draining the bladder and other parts of the urino-genital system. In addition, this parasite is dioecious, long lived and reproduces by depositing eggs in the capillary plexuses of the bladder epithelium. The eggs are hard shelled with a terminal spine. They secrete a complex substance, the soluble egg antigen, that stimulates local inflammation which assists passage through the urothelium and the eggs are passed out in the urine. If the eggs are deposited in freshwater then they hatch a free swimming larva which infects specific freshwater snails to continue the life cycle. Over time and particularly in adult life severe hyperplasia occurs in the bladder wall, lesions form and cancerous tissue can develop [1].Children frequently become infected through domestic chores or recreational contact with water. This too can become debilitating, but most lesions will clear following treatment [2]. Among adults who are infected, much of the pathology depends on their life style and occupational activities. In rural areas, large portions of the community are involved in agricultural activity and to a great extent their contact with water from river systems will dictate the nature and chronicity of the infections and the subsequent damage to the urogenital organs. In adults with long term infection of the bladder and urinary tract, there can be dire consequences-the extent of which is often difficult to assess [3].

Diagnosis of urogenital schistosomiasis in adult patients is quite challenging especially in chronically infected adults who are often misdiagnosed when current diagnostic methods are employed, because they pass few schistosome eggs in their urine. In general, the performance of current diagnostic procedures in both children and adults, is variable and the significant inconsistencies of their sensitivities (Se) and specificities (Sp) have made it difficult to set a gold standard because of both age related and epidemiological differences in schistosomiasis prevalence and egg passage [4].

Detection of haematuria in urine, which has been proposed as a valid indication of schistosome infection, has been widely adopted in many national schistosomiasis control programmes [5], [6] It is inexpensive, easy to use in field conditions and quick to assess infection. However haematuria is a sign of infection as well as other conditions and it is not a definitive test. Misdiagnosis is exacerbated when adults are involved or when prevalence of infection is low. Under these conditions, infections may be chronic but causing severe lower urinary tract disease; they may be of low intensity with sporadic egg passage that can be missed in single specimens, but under both circumstances the diagnosis of infection is definitely problematic [4] and in the clinical condition it frequently becomes essential to obtain a diagnosis [7]. It may cause severe life threatening disease in the bladder or kidney or in the case of children a means of maintaining transmission into snail infested water. In order to deal accurately with these specific situations, a definitive method of diagnosis of schistosomiasis is required because detection of parasite eggs in excrement is insufficiently sensitive.

Detection of fragments of pathogen associated DNA in urine has been demonstrated [8] and to this end, schistosomes lend themselves ideally. The pioneering work of Hamburger and others [9] has shown the extensive nature of the *S. haematobium*-specific *Dra*1 repeat fragment which they demonstrated in cercariae and in the adult form. We have recently shown that this fragment can be detected with high Se and Sp in urine of infected people both those passing eggs of the parasite as well as in several individuals, both adult and children, in whose urine no eggs were detected [10]. Furthermore, we demonstrated that urine based DNA could be extracted from filter paper through which 50 ml of urine was filtered and dried thus introducing a simple field collection system. Nevertheless there remains an important requirement which is to subject the available diagnostic procedures to rigourous comparison and evaluate and indicate the most effective method.

Latent Class (LC) models have been recommended as a statistical procedure that can be used to obtain direct estimates of Se and Sp in the absence of an acceptable gold standard. This analytical process was used, among others for *S. mansoni* [11], *S. japonicum* [12] and hookworm [13], as well as by Koukounari et al [4] for *S. haematobium* to compare a series of five diagnostic procedures on adult volunteers recruited from three villages in Ghana. None of these tests involved the use of PCR and detection of *Dra*1, and the results were equivocal with haematuria and detection of eggs in urine being the best predictors of infection, but neither being clinically acceptable. Since infections must be correctly diagnosed for effective disease control [6], and the accuracy of current methods has been previously challenged, the quest for an effective test is warranted.

The main aim of this study was to evaluate several tests for the detection of *S. haematobium* infection- including urine based DNA- in adults through LC models and by using adults' data from Western Nigeria.

## Materials and Methods

### Study sites

Samples were collected from consenting inhabitants in six rural villages in Ogun State, Western Nigeria. The villages, Apojola, Igbole, Lopokoredi, Akande, Sasa and Ogbere located in two local government areas (Odeda and Ijebu-East) were selected based on the results of the survey conducted by the Nigerian Federal Ministry of Health as part of schistosomiasis control programme in 2009 [14]. According to the survey, these areas are endemic for *S. haematobium* with no reported cases of *S. mansoni* infection. Apart from Ogbere (pop 15,000) in each of the other villages (pop 1,000 to 3,000) people are occupied in variegated agricultural practices.

### Data and urine sample collection

The aforementioned villages were visited systematically to enable the process of informed consent to take place. This process complied with institutional approvals obtained from both Johns Hopkins SPH Institutional Research Board (ref IRB00002920) and Ethical and Research Committee in Obafemi Awolowo University (ref 0004553). Initially, 435 adults between the ages of 20 and 55 years were enrolled in the study, of which 401 provided complete data (when the ‘age’ and ‘sex’ variables were not considered). Volunteers were recruited to school halls in each village and a record form bearing the age, gender, and occupation of unidentified individuals was administered. There was no history of anti-schistosome treatment in any of the villages.

Following oral consent, volunteers were assigned identification numbers which were applied to 200-ml plastic containers provided. They were asked to give as much urine as possible. Urine was collected between 10:00h and 14:00 for optimum egg passage [15]. Each urine sample was tested with a Hemastix® (Bayer,Corp. Elkhart. Indiana. USA) test strip to detect haematuria. Those who were haematuric were provided treatment at local government clinics under professional care of a certified nurse. Approximately 50 ml urine specimens collected were swirled and filtered through 12.5 cm Whatman No. 3 filter papers, (GE Healthcare. Bucks. UK) folded in a cone. Each paper was numbered and marked to show the portion exposed to urine. The papers were dried under fly proof cover transported to the laboratory and processed for parasitological (detection of eggs) and molecular (detection of *S. haematobium*-specific DNA) examination [10].

### Presence of parasite eggs

The procedure for microscopic examination has been described previously [10]. Briefly, the central 2 cm portion of each filter paper was excised and divided into quadrants. Only the portion exposed to the urine was used. One marked quadrant was stained with ninhydrin solution (0.2% ninhydrin in 70% ethanol) and allowed to develop in the dark for at least 15 min. and eggs were counted using a dissecting microscope. Obviously this could be considered a semi-quantitative method, but it was not intended to compare with the standard nucleopore filtration of 10 ml urine.

### PCR amplification

For PCR assay, water extraction of DNA from filter papers was carried out as follows: The other marked quadrant of the 2 cm disc was cut in two, and one segment was sliced into 2–3 mm squares. This was immersed in 600 µl nuclease-free water in a 1.5 ml eppendorf tube, incubated at 95°C for 10 min and shaken at room temperature overnight (12 hrs). The paper was removed and the tube centrifuged at 3,000 rpm for 10 min. DNA was then precipitated and concentrated using the Qiagen® QIAmp (Qiagen Maryland, USA) mini- kit according to the manufacturer's protocol. The extracted DNA was used for PCR amplification. Sh primers were designed to amplify the 121 bp tandem repeat *Dra*-1 sequence of *S. haematobium* as described by Hamburger [9]. Samples were run in 25 ul volumes comprising 1.25 units Taq DNA polymerase, 2.5 µL 10× buffers, 1.5 mM MgCl_2_, 200 µM (each) of dATP, dCTP, dGTP, and dTTP, 1 µM of each of the amplification primers and 5 µL of template DNA. Annealing cycles consisted of denaturation at 95°C for 15 min, followed by 33 cycles of 95°C for 30 sec, annealing at 53°C for 1.5 min and extension at 72°C for 1 min. The final extension step was at 60°C for 5 min. Products were analyzed on a 2% agarose gel stained with ethidium bromide (1 µg/ml) and visualized with ultraviolet light.

### Statistical analysis

Since we did not want to consider any of the three diagnostic tests used in this study as a perfect reference test, we obtained estimates of Se and Sp for each of these three examined diagnostic tests by latent class analysis (LCA) [16]. LCA is a statistical modeling technique which examines associations between observed variables (in our case the three examined diagnostic tests: dipstick for haematuria, microscopy and PCR) that imperfectly measure a non-observable (latent) variable. In the current study, we consider the true *S. haematobium* infection status of a sample of Nigerian adults to be a latent variable with two categories: ‘infected’ and ‘non-infected’. In other words, we considered the observed data of the three diagnostic tests as indicators of an underlying, not directly observable variable (i.e. *S. haematobium* infection). The modeled associations are induced by those latent variables' underlying constructs.

The observed binary variables (*x_1j_*, *x_2j_*, and *x_3j_*) were defined such that *x_ij_ = 0* represents a negative result for diagnostic test *i* and *x_ij_ = 1* represents a positive test result for diagnostic test *i* on individual *j*. We tested whether correlations between these observed variables could be accounted for by a single latent dichotomous variable *Y* (i.e. the absence *Y* = 0 or presence *Y* = 1 of *S. haematobium* infection) and we defined *η = P(Y = 1)* the probability of being in the infected latent class. In other words, we divided the studied population into two classes (i.e. non infected and infected) assuming that the *x_ij_'*s were mutually independent within each class (i.e. true infection status). This is known as the conditional or local independence assumption in LCA which affirms that the results from the three diagnostic tests in the same individual were independent within the real condition of illness. We tested this assumption by speculating the standardized residuals for each response pattern from the 3 diagnostic tests as estimated from the LCA model [17].

The likelihood function of the LC model for a random sample of N individuals is
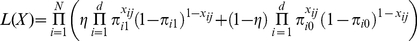
(1)Such a model has two types of parameters. First, there is the unconditional probability *η* that a person is in the infected latent class. The second type of parameters are the conditional probabilities *π_i1_* and *π_i0_* that an individual in a particular latent class has a specified value of each of the manifest variables [18]. In fact, *π_i1_* represents the Se and is the conditional probability *P(x_i_ = 1|y_j_ = 1)* while *(1−π_i0_)* represents the Sp and is the conditional probability *P(x_i_ = 0|y_j_ = 0)*. The LC model hence produces an estimate of disease prevalence as *η* is the proportion of individuals in the population of which our sample is expected to be in infection class *Y = 1*. It also provides direct estimates of Se and Sp for all the diagnostic tests [19].

Conclusively, LCA models the probability of each combination of diagnostic tests results (or response pattern) as a weighted sum of the conditional probabilities of a result of the diagnostic test given the ‘true’ infection status (i.e. latent class). The weights are the unconditional probability of a randomly selected individual being allocated in one of the two latent classes.

We also employed multigroup LCA by including grouping variables and examining group differences of measurement invariance. In this study such group differences were examined for males/females and different age groups (it should be noted here that such groups represent distinct populations). Likelihood ratio tests between less and more restrictive models were used to examine differences in infection prevalence and measurement invariance as well as partial measurement invariance between groups. For instance, a significant measurement invariance tests suggests that Se and Sp of the diagnostic tests vary by group and should be estimated for each group without specifying for which diagnostic test this could be the case and thus the hypothesis of partial measurement invariance was also examined wherever applicable.

We also employed LCA with covariates such as occupation and village location in order to examine differences in *S. haematobium* infection prevalence between these different groups. Such an approach starts with the idea that there is a single population of research participants, but the participants vary in terms of latent class membership-i.e. their *S. haematobium* prevalence varies-and this variance can be explained by one or more covariates. For all analyses presented in this study we used MPLUS 6.1 [20].

## Results

Table 1 represents the observed positive results expressed as percentages of *S. haematobium* infection for the three diagnostic tests stratified first by sex and then by age groups. Different diagnostic tests gave different proportions of positive results for both males and females as well as different age groups. Table 2 shows urogenital infection as determined by microscopy and PCR. There were 0 people that were negative by PCR and positive by microscopy. Only 43 out of the 401 (10.7%) had discordant results (they were positive by PCR and negative by microscopy). Tables 3 and 4 refer to LC models with grouping variables such as sex and age groups when partial measurement invariance was considered.

**Table 1:** Positive results (percentages) by for the three diagnostic tests stratified by sex and age groups.

Variable	Adults No (%)	Positive results by Haematuria % (95% CI)	Positive results by PCR (95% CI)	Positive results by Microscopy (95% CI)
Sex (n = 397)				
Male	172 (43.3)	44.19 (36.76 to 51.61)	48.84 (41.37 to 56.31)	30.23 (23.37 to 37.10)
Female	225 (56.7)	40.00 (33.60 to 46.40)	26.67 (20.89 to 32.44)	21.78 (16.38 to 27.17)
Age (n = 399)				
25–35 years old	212 (53.1)	49.06 (42.33 to 55.79)	39.62 (33.04 to 46.21)	32.08 (25.79 to 38.36)
36–45 years old	67 (16.8)	35.82 (24.34 to 47.30)	37.31 (25.73 to 48.89)	25.37 (14.95 to 35.79)
46–55 years old	120 (30.7)	30.83 (22.57 to 39.10)	28.33 (20.27 to 36.40)	12.50 (6.58 to18.42)

**Table 2:** 2×2 descriptive table for schistosomiasis infection status as determined by PCR and microscopy.

	Microscopy		
PCR	−	+	Total
−	257	0	257
+	43	101	144
Total	300	101	401

**Table 3:** ‘LC Model 1’(n = 397) with partial measurement invariance for gender.

Haematuria			Microscopy		PCR	
	Sensitivity	Specificity	Sensitivity	Specificity	Sensitivity	Specificity
Males	87.6%(p = 0.001)	34.7%(p = 0.871)	70.1%(p = 1.000)	100%(p<0.001)	100.0%(p = 1.000)	100.0%(p = 1.000)
Females	86.7%(p<0.001)	77.0%(p<0.001)				
Prevalence of *S. haematobium* infection						
Males	48.83%(p<0.001)					
Females	26.66%(p<0.001)					

**Table 4:** ‘LC Model 2’ (n = 399) with partial measurement invariance for age groups.

Haematuria			Microscopy		PCR	
	Sensitivity	Specificity	Sensitivity	Specificity	Sensitivity	Specificity
Age category(25–35 years old)	84.5%(p<0.001)	74.2%(p = 0.001)	69.9.0%(p = 1.000)	100.0%(p<0.001)	100.0%(p = 1.000)	100.0%(p = 0.996)
(36–45 years old)	85.0%(p = 0.949)	38.9%(p = 0.017)				
(46–55 years old)	74.3%(p = 0.345)	38.3%(p = 0.001)				
Prevalence of *S. haematobium* infection						
(25–35 years old)	39.63%(p = 0.003)					
(36–45 years old)	37.31%(p = 0.736)					
(46–55 years old)	38.33%(p = 0.040)					

Likelihood ratio tests between less and more restrictive models indicated differences in infection prevalence and measurement invariance between groups (i.e. when sex and age groups are considered separately in a multiple-group model approach). Such results are not presented here. More precisely, the significant measurement invariance tests suggested that Se and Sp of the diagnostic tests varied by groups and should be estimated for each of these groups. Also the rejection of the null hypothesis of measurement invariance (i.e. that we found that Se and Sp of the three diagnostic tests finally varied by groups) implied that simply the performance of the diagnostic tests differs among groups, without specifying for which diagnostic test this could be the case. Therefore, we also examined the hypotheses of partial measurement invariance in order to establish for which diagnostic tests their performance might vary among groups.

Specifically, Table 3 represents the results of one LC model as it was dictated by likelihood ratio tests and where partial measurement invariance was found to hold when gender was considered in a multiple-group model approach. This model, denoted in table as ‘LC Model 1’ is an LC model where Se and Sp for microscopy and PCR were identical among males and females but they were varying by gender for the haematuria test. More precisely, haematuria was found to be equally sensitive and more specific for females compared to males. Microscopy was found to be the less sensitive diagnostic test (Se = 70.1%) if compared with haematuria (Se = 87.6% for males and 86.7% for females) and PCR (Se = 100.0%).

We also tested if the estimated *S. haematobium* prevalence for males and females as derived from the LC Model1 –shown in Table 3- was affected by the partial measurement invariance by comparing the estimated *S. haematobium* prevalence for males and females as derived from the fully restricted model i.e. the model where Se and Sp did not vary by gender for any examined diagnostic test. (such results are not shown here). Such measures were found similar between the two models. In particular, it is estimated that the prevalence of *S. haematobium* infection was highest among males (48.83%) compared to females (26.66%).

Table 4 represents the results of one LC model as it was dictated by likelihood ratio tests (such results are not presented here) and where partial measurement invariance was found to hold when age groups were considered in a multiple-group model approach. This model and denoted in table as ‘LC Model 2’ is an LC model where Se and Sp for microscopy and PCR were identical among different age groups but they were varying by age category for the haematuria test. More precisely, haematuria was found to be less specific in the older age groups (Sp = 38.9% for the 36–45 years old and Sp = 38.3% for the 46–55 years old) than in the younger one (Sp = 74.2% for the 25–35 years old). Microscopy was found to be again the less sensitive diagnostic test if compared (Se = 69. 9%) with haematuria (where the Se was estimated to be greater than 70.0% for all age groups) and PCR (Se = 100.0%). Similarly as before, by following the same methodology, *S. haematobium* prevalences were not found to be affected by the partial measurement invariance once age groups were considered in the multiple-group model approach. It is estimated that the prevalence of *S. haematobium* infection was highest for the younger age group (39.63%) compared to older age groups (37.31% for the 36–45 years old and 28.33% for the 46–55 years old).

Covariate model approach (dummy-coded grouping variable) for the variables ‘occupation’ and ‘village location’ permitted examination of differences in *S. haematobium* infection prevalence between these different groups. *S. haematobium* prevalence was significantly different among different occupation groups. In particular *S. haematobium* prevalences were estimated from the LC model to be 64.29% (p - value = 0.035) in fishermen; in those individuals occupied with cattle rearing *S. haematobium* prevalence was 50% (p - value = 0.496); in housewives this was 46.68% (p-value = 0.220); in those individuals occupied in farming this was 30.11% (p-value<0.001); in those involved in teaching this was 30.01% (p-value = 0.054). *S. haematobium* prevalence varied significantly among different village locations; for instance highest *S. haematobium* prevalences were found in Apojola (prevalence = 57.25%, p-value = 0.090), Lopokoredi (prevalence = 43.93%, p-value = 0.076), Akande (prevalence = 32.45%, p-value = 0.001) as well as Igbole (prevalence = 30.77%, p-value = 0.078).

Standardized residuals for each response pattern from the 3 diagnostic tests from the invariant LC model with 2 classes were all almost between -2 and 2 which indicates a good model fit and that the assumption of local independence of the 3 diagnostic tests, holds here (this is the starting LC model-not presented here-before multigroup analysis for testing measurement invariance, takes place).

To test the effect of parasite clearance on the persistence of *Dra*1 fragment, a group of 55 persons who were positive either for presence of parasites eggs and/or DNA in the urine, were followed up two weeks after treatment with praziquantel. Of these 26 (47.3%) were positive for parasite eggs and 42 (76.4%) were positive for DNA. None of these people were positive for eggs or parasite specific DNA in the follow up.

## Discussion

The recruitment process we employed was designed to acquire samples with sufficient diversity to enable a thorough, rigourous comparison of three test procedures across males and females and enable stratification in both age and exposure patterns of S. *haematobium* infection. The sample of approximately 400 volunteers between 20 and 55 years were recruited *ad hoc* from six separate villages where data from the State Ministry of Health indicated the presence of *S. haematobium* infections at differing intensities. We compared a series of LC models under all these conditions before final results and conclusions would be drawn. All LC models identified the detection of the *Dra*1 DNA as a consistent and definitive diagnosis. This does not negate the role of haematuria as a sign of schistosome infection or detection of parasite eggs as a diagnostic, but it provides a secure additional test for specific use.

Definitive diagnosis requires the demonstration of the causative agent, a requirement that, while ideal, may not be feasible. With schistosomiasis this has been a continual problem and in clinical cases rectal biopsy is recommended to improve diagnosis [7], however such invasive procedures are not a satisfactory option. When considering *S. haematobium* and because the standard treatment- praziquantel- is a well tolerated drug, it is ethical to resort to detection of haematuria as a sign of infection, particularly in children and in mass chemotherapy programmes. As we have demonstrated, among adults, particularly in the older age groups haematuria has low specificity (38%) and thus it is not an adequate test. Microscopic detection of parasite eggs also is deficient in these age groups where the Se is low (<70% in Table 2). Such results have important epidemiological and clinical implications. For instance, epidemiological studies based on egg detection tend to discount the importance of adults as reservoirs of the parasite population and thus concentrate on children for targeted control. In a recent study we have demonstrated an almost two-fold difference in prevalence of *S. haematobium* in adults when comparing egg detection with presence of parasite DNA [10]. Undoubtedly, we need a more reliable test in order to assess accurately the status of the untreated reservoir in control operations. Additionally, in adult populations, and in particular in the clinical situation where severe damage of the bladder is suspected, a definitive diagnosis is necessary.

Expenses are incurred in performing PCR for diagnosis, but the procedure is becoming common in most parts of the world where schistosome parasites exist and its availability is not an impediment when needed. While PCR procedures are often discounted as field operations, it is important to reconsider this when the merits of the diagnostic test are demonstrated, as we have shown in the current study. In fact in most developing countries facilities for PCR do already exist and are operational thus enabling the integration of improved methodologies into the field.

A diagnosis of *S. haematobium* is often needed in clinical conditions. In cases where bladder lesions are detected with ultrasound, it is necessary to know if this parasite is present even before invasive biopsy is indicated. In field control situations, there is a need to detect low level infections even in children, where infection occurs in the absence of eggs or haematuria [21]. These cases although silent can still infect snails and perpetuate the cycle. In both situations a simple urine specimen, filtered through a filter paper can be used for DNA detection, and PCR procedure will give a definitive result.
